# Hypersensitivity pneumonitis: the importance of the radiologist in the multidisciplinary approach to its diagnosis

**DOI:** 10.1590/0100-3984.2016.49.2e2

**Published:** 2016

**Authors:** Luiz Felipe Nobre, Leila John Marques Steidle

**Affiliations:** 1 PhD, Adjunct Professor of Radiology and Pulmonology in the Department of Clinical Medicine, Universidade Federal de Santa Catarina (UFSC), Florianópolis, SC, Brazil.

Hypersensitivity pneumonitis encompasses a set of diseases with pulmonary involvement and a predominance of histopathologic findings-lymphocytic inflammatory infiltrate, noncaseating granulomas, and foci of bronchiolitis obliterans, as well as fibrosis in the more chronic stages^(1-3)^-predominantly distributed around the small airways, as shown in the excellent correlation study conducted by Torres et al.^(4)^, published in this issue of **Radiologia Brasileira**. That distribution of findings reflects a response to repeated inhalation of various antigenic substances, typically organic substances, such as fungi, thermophilic bacteria, and bird feathers^(1,2)^. Farmer's lung and bird fancier's lung are well-established forms of hypersensitivity pneumonitis. However, new and curious forms are described every day, such as "saxophonist's lung", which is caused by exposure to molds that grow in the mouthpieces of saxophones.

Clinically, hypersensitivity pneumonitis can present in acute, subacute, or chronic forms. The acute and subacute forms have systemic symptoms, mimicking those of flu or asthma. The chronic form occurs in individuals with greater re-exposure to antigens, evolving to more pronounced interstitial fibrosis, dyspnea, hypoxemia, digital clubbing, and impaired lung function. The chronic form is also usually associated with failure to identify the causative antigen, and the differential diagnosis includes other idiopathic interstitial lung diseases^(1,5)^, although making that distinction is often impossible even with the histopathological findings.

There is considerable variation across epidemiological studies of hypersensitivity pneumonitis. It is estimated that hypersensitivity pneumonitis accounts for 3-13% of all cases of interstitial lung disease in Brazil^(3)^. A study conducted in the city of São Paulo, SP, Brazil, involving 99 cases of lung biopsy-confirmed hypersensitivity pneumonitis, indicated that the most frequent causative agents are household mold and bird droppings^(6)^. In our experience in the Brazilian state of Santa Catarina, we have also found that the disease occurs most frequently in individuals exposed to mold in the home and in individuals who are bird breeders.

There are no established criteria for the diagnosis of hypersensitivity pneumonitis. In practice, the fundamental components are a correlation between the clinical and functional history consistent with the diagnosis, together with known exposure, marked lymphocytosis in the bronchoalveolar lavage fluid, characteristic biopsy findings, and evidence of the disease on a high-resolution computed tomography scan of the chest^(1,3,7)^.

The removal of the source of the antigen exposure is critical for the treatment of hypersensitivity pneumonitis. Some authors have attempted to draw attention to the importance of mold exposure in the home, a cause of hypersensitivity pneumonitis that is without a doubt widely underestimated. A detailed inspection of the environment is the most important step in determining the specific source of the exposure and in developing control strategies.

A multidisciplinary approach is essential to making the definitive diagnosis of hypersensitivity pneumonitis, as well documented by Torres et al.^(4)^, and establishing a correlation among the clinical characteristics, the causal nexus of the exposure, tomography findings, and histopathological aspects is fundamental.

In the context of hypersensitivity pneumonitis, computed tomography plays a fundamental role in demonstrating findings suggestive of the disease. Although not pathognomonic, these findings should suggest the possibility of hypersensitivity pneumonitis in the differential diagnosis, and the radiologist who is attentive to and knowledgeable regarding such findings should always investigate the patient history of respiratory exposure, in order to determine whether it is consistent with the diagnosis. Especially in the acute and subacute forms of the disease, the findings of ground-glass centrilobular nodules and a mosaic pattern of attenuation without vascular redistribution should raise the suspicion of hypersensitivity pneumonitis. In the acute form, the main differential diagnosis is with smoking-related respiratory bronchiolitis, which facilitates the diagnostic reasoning, because hypersensitivity pneumonitis rarely occurs in smokers. In the subacute form, the main differential diagnosis is nonspecific interstitial pneumonia, which is also often associated with various clinical symptoms of hypersensitivity pneumonitis. As also discussed in the Torres et al. article^(4)^, the presence of focal hyperinflation of secondary pulmonary lobules interspersed among the diffuse lung alterations supports the suspicion of hypersensitivity pneumonitis, as evidenced by cellular or constrictive bronchiolitis secondary to the bronchiolocentric changes. The presumptive diagnosis of hypersensitivity pneumonitis can be made in the radiology department, on the basis of a tomography finding suggestive of an exposure history consistent with the disease, which can go unnoticed if not addressed in his-tory-taking process. Therefore, it is one of those diseases in which radiologists can increase their credibility with their colleagues in other specialties. A critical diagnostic suggestion, based on an indicative image, can make a radiologist famous!
